# Reaction Dynamics of Flavonoids and Carotenoids as Antioxidants

**DOI:** 10.3390/molecules17022140

**Published:** 2012-02-21

**Authors:** Rui-Min Han, Jian-Ping Zhang, Leif H. Skibsted

**Affiliations:** 1 Department of Chemistry, Renmin University of China, Zhongguancun Street, No. 59, Haidian District, Beijing, 100872, China; 2 Food Chemistry, Department of Food Science, University of Copenhagen, Rolighedsvej 30, DK-1958 Frederiksberg C, Denmark; Email: ls@life.ku.dk

**Keywords:** flavonoids, carotenoids, antioxidant synergism, antioxidant antagonism, free radical kinetics

## Abstract

Flavonoids and carotenoids with rich structural diversity are ubiquitously present in the plant kingdom. Flavonoids, and especially their glycosides, are more hydrophilic than most carotenoids. The interaction of flavonoids with carotenoids occurs accordingly at water/lipid interfaces and has been found important for the functions of flavonoids as antioxidants in the water phase and especially for the function of carotenoids as antioxidants in the lipid phase. Based on real-time kinetic methods for the fast reactions between (iso)flavonoids and radicals of carotenoids, antioxidant synergism during protection of unsaturated lipids has been found to depend on: (i) the appropriate distribution of (iso)flavonoids at water/lipid interface, (ii) the difference between the oxidation potentials of (iso)flavonoid and carotenoid and, (iii) the presence of electron-withdrawing groups in the carotenoid for facile electron transfer. For some (unfavorable) combinations of (iso)flavonoids and carotenoids, antioxidant synergism is replaced by antagonism, despite large potential differences. For contact with the lipid phase, the lipid/water partition coefficient is of importance as a macroscopic property for the flavonoids, while intramolecular rotation towards coplanarity upon oxidation by the carotenoid radical cation has been identified by quantum mechanical calculations to be an important microscopic property. For carotenoids, anchoring in water/lipid interface by hydrophilic groups allow the carotenoids to serve as molecular wiring across membranes for electron transport.

## Abbreviations

**ABTS**: 2,2'-Azino-bis(3-ethylbenzothiazoline-6-sulfonic) acid
**AMVN**: 2,2'-azobis(2,4-dimethylvaleronitrile)
**ANS**: 8-anilino-1-naphthalenesulfonic acid
**16-AP**: 16-(9-anthroyloxy) palmitic acid
**AscH^−^**: ascorbate
**Car**: carotenoid
**β-Car**: β-carotene
**DPPC**: dipalmitoyl phosphatidyl choline
**EC**: (−)-epicatechin
**EGC**: (−)-epigallocatechin
**ECG**: (−)-epicatechin gallate
**EGCG**: (−)-epigallocatechin gallate
**ET**: electron transfer
**HAT**: hydrogen atom transfer
**N-HPT**: N-hydroxypyridine-2(1H)-thione
**PC**: phosphatidyl choline
**RAF**: radical adduct formation
**ROS**: reactive oxygen species
**SAR**: structure-activity relationship
**TOH**: tocopherol

## 1. Introduction

Flavonoids and carotenoids are naturally occurring pigments ubiquitously present in the plant kingdom and in other types of photosynthetic organisms, playing important roles in light harvesting, photo-protection and antioxidation [1,2]. They are also exogenous antioxidant compounds for animals and humans through daily consumption of a diet of grains, vegetables and fruits [3,4,5,6,7].

In metabolic processes, oxidation reactions involving electron transfer yield energy powering aerobic life, but concomitantly produce aggressive free radicals. Oxygen-centered free radicals, such as superoxide (O_2_^•–^), peroxyl (ROO^•^), alkoxyl (RO^•^) and hydroxyl (^•^OH), and nitric oxide (NO^•^), when formed in excessive amount, may exert oxidative stress to biological systems, and proteins, lipids and DNA may be damaged [8,9]. Oxidation stress has been associated with aging and with certain degenerative diseases of the cardiovascular system, cataracts, cognitive dysfunction and cancer [10,11,12]. Certain vitamins constitute a well-established category of exogenous antioxidants together with carotenoids and phenolic compounds like the flavonoids supporting the endogenous antioxidative systems.

A qualified antioxidant should undertake one or more of the following actions in order to protect against oxidative damage of biological systems [13,14,15]: (i) oxygen depletion; (ii) quenching of singlet oxygen; (iii) chelation of metal ions which otherwise could catalyze formation of reactive oxygen species (ROS); (iv) scavenging of ROS or termination of chain reaction of oxidation propagation; or (v) repairing of oxidative damage. Among these mechanisms the essential functions as antioxidant are to deactivate reactive oxidants such as singlet molecular oxygen (^1^O_2_*) and ROS. Flavonoids and carotenoids fulfill most of these functions, depending on their individual structure and become accordingly important as non-vitamin antioxidants.

**Scheme 1:**
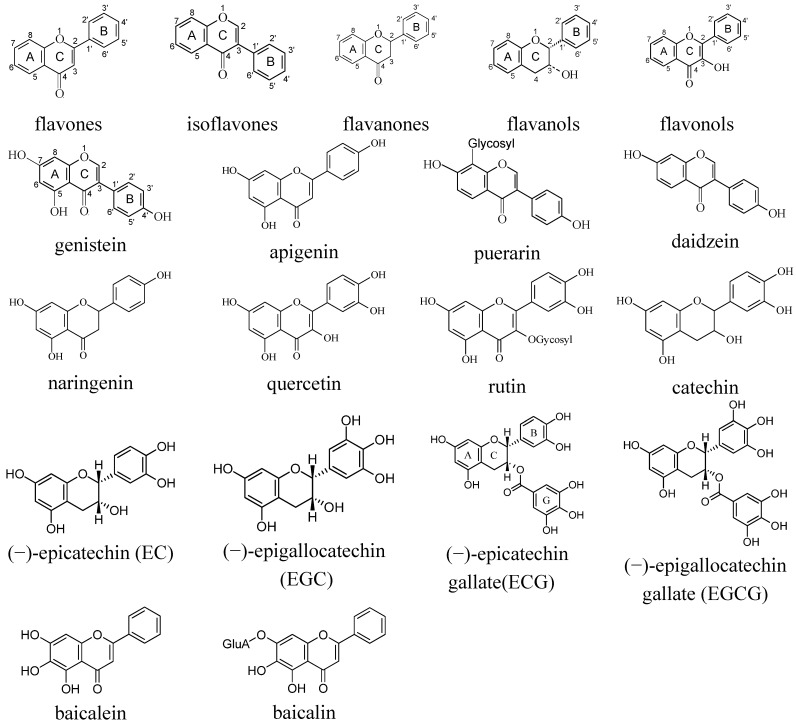
Backbone structures of flavone, isoflavone, flavanone, flavanol, and flavonol (*upper*), and molecular structures of representative (iso)flavonoids (*lower*).

Flavonoids are polyphenolic substances present in most plants with a rich structural diversity, as more than 8,000 flavonoids have been characterized [16]. Flavonoids and their glycosides act as hydrophilic antioxidants, antimicrobials, photoreceptors, visual attractors, feeding repellants, and as UV-light filters and substrate for polyphenoloxidases protecting tissue after physical damage to plants. It is reported that flavonoids might account for at least part of the health benefits associated with vegetable and fruit consumption. Flavonoids are built upon a C6-C3-C6 flavone skeleton in which the two aromatic rings are linked by three carbons cyclized with oxygen. Several classes of flavonoids differ with respect to the degree of unsaturation and oxidation of the three-carbon segment, as shown with a few representative structures in Scheme 1. 

**Scheme 2:**
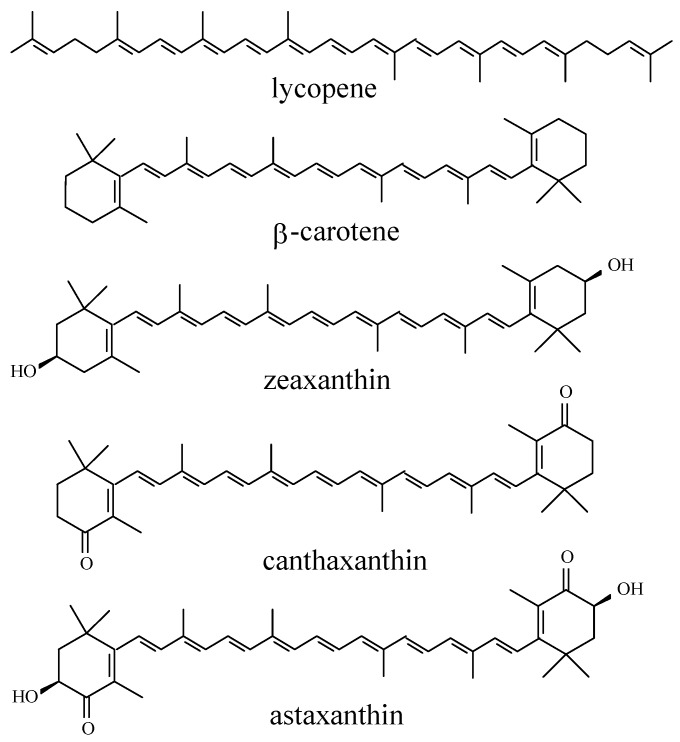
Molecular structures of C_40_-carotenoids. The binding of the molecules at the water/lipid interface is considered important for the antioxidant efficiency

Carotenoids are lipophilic, but the xanthophylls, which are carotenoids with polar hydroxyl and keto functionalities, as seen in Scheme 2, have increased affinities for lipid/water interfaces. The number of naturally occurring carotenoids has been reported to reach about 750 [17]. Carotenoids have important functions in animals as colorants and in relation to vision and for protection of sensitive structures as in eggs during hatching and are specifically up-concentrated in relevant tissue from dietary sources.

Reactivity and fate of any antioxidant radical resulting from scavenging of radicals involved in initiation and propagation of lipid and protein oxidation always need to be characterized since antioxidant radicals hold the potential of initiating other oxidative reactions or of depleting other biologically important antioxidants [18,19,20,21]. The carotenoid-flavonoid interaction is of special interest, because in biological or food systems different types of exogenous antioxidants co-exist, and the issue of synergistic, additive or antagonistic interaction is obviously important. Particularly, the antioxidative interactions between carotenoid and flavonoid have not been examined in any detail. To these ends, the molecular and electronic structures, the physicochemical properties of the antioxidant and its free radicals, as well as the effects from microenvironments have been examined for a number of structurally related hydrophilic flavonoids (Scheme 1) and lipophilic carotenoids (Scheme 2). It is worth noting that in systems of increasing organization, *i.e.*, from homogenous aqueous solutions or edible oils to structured emulsions, liposomes, foods or other biological systems, mechanistic investigation of antioxidative interaction becomes more intricate. Therefore, at the present stage organic or aqueous solutions were selected as homogenous model systems while liposomes were used as heterogeneous model systems in order to elucidate the structure-activity relationship (SAR) of these groups of antioxidants. The use of various optical spectroscopic techniques to detect radical products of flavonoids and carotenoids has established a methodology, which is potent in probing fast reactions and which moreover is noninvasive for future *in vivo* detection of radicals when combined with optical microscopy. In order to discuss the potential of this approach, the reaction dynamics of formation of radicals of flavonoid and carotenoids in homogeneous solution will be described together with the relation to the activity as antioxidant of flavonoids and carotenoids (Section 2). The carotenoid-flavonoid interactions during radical scavenging and in anti-lipooxidation will be summarized (Section 3). In addition, a discussion of the future trend along this research line will be given (Section 4).

## 2. Radical Scavenging of Flavonoids and Carotenoids

### 2.1. Flavonoids

Due to their low redox potentials (0.2 < *E*^0^ < 0.8) [22], flavonoids are thermodynamically able to reduce most oxidizing free radicals relevant to biological systems such as superoxide, peroxyl, alkoxyl, and hydroxyl radicals.

*Radical scavenging.* Antioxidant capacity assays have mainly classified flavonoids as scavenging by electron transfer (ET) or by hydrogen atom transfer (HAT) [23,24]. Generally, there are two possibilities for phenolic antioxidants reacting with free radicals (R^•^): (i) one-step hydrogen atom transfer as in the reaction of Equation (1), or (ii) electron transfer followed by proton transfer as shown in the reaction of Equation (2). At higher pH, where phenols are deprotonated, only the electron transfer mechanism is possible, as shown in the reaction of Equation (3). A phenolate form has a higher radical scavenging potential than its parent phenolic form owing to a lower redox potential. For most scavenging reactions of the phenol itself it is unclear whether the reaction between the phenolic antioxidant and the reactive radical proceeds via one-step hydrogen transfer or via an initial electron transfer.


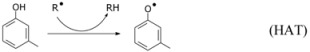
(1)


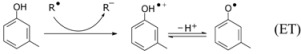
(2)


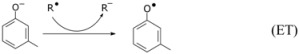
(3)

**Scheme 3:**
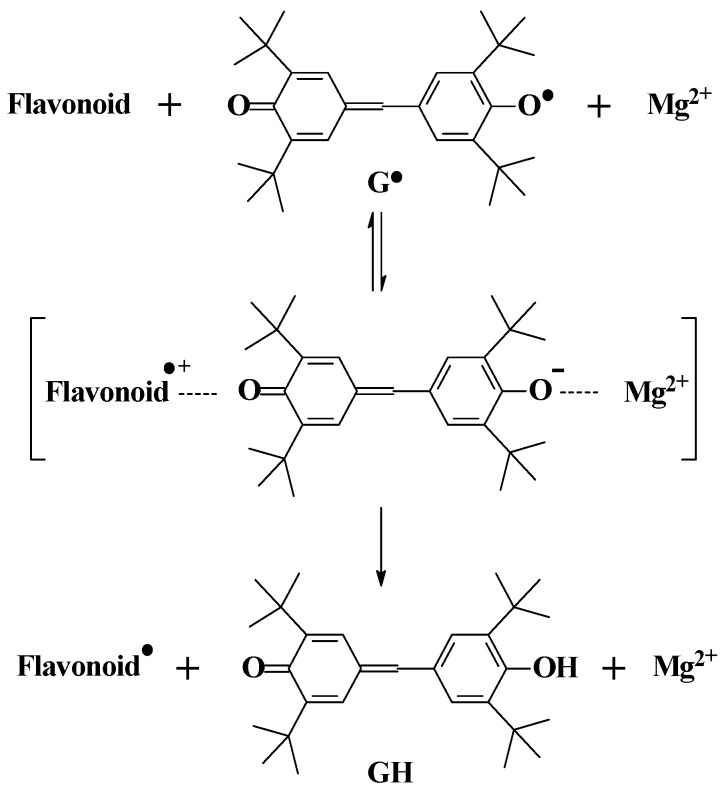
Hydrogen and electron transfer mechanisms of flavonoids. Acceleration of radical scavenging of galvoxyl radical (G^•^) by the presence of magnesium (II) as rate-determining is indicative of electron transfer [25].

A criterion for distinguishing between hydrogen and electron transfer has been developed using magnesium(II) and the galvinoxyl radical (G^•^) as shown in Scheme 3 [25]. If the scavenging of G^•^ by a phenolic antioxidant involves an electron transfer as the rate determining step, the rate of scavenging is accelerated by the presence of the Mg^2+^ ion due to stabilizationof the one-electron reduced anion of the radical. The initial electron transfer is followed by a proton transfer from the antioxidant radical cation to G^−^ to yield antioxidant neutral radical and GH. Using this method, (+)-catechin was confirmed to scavenge the galvinoyl radical via electron transfer followed by proton transfer rather than via direct one-step hydrogen atom transfer.

*Stability of flavonoid radicals.* ROS-scavenging potential of flavonoids has been related to the stability of their radical species, and the stability of flavonoid radicals is expected to increase by extended conjugation. The stability of flavonoid radicals is most easily investigated after the parent flavonoids reacting with reactive radicals generated by pulse radiolysis or by laser flash photolysis, or through direct photoexcitation to the singlet excited states (S_1_) of neutral or anionic flavonoids:


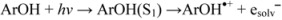
(4)


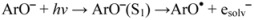
(5)

**Scheme 4:**
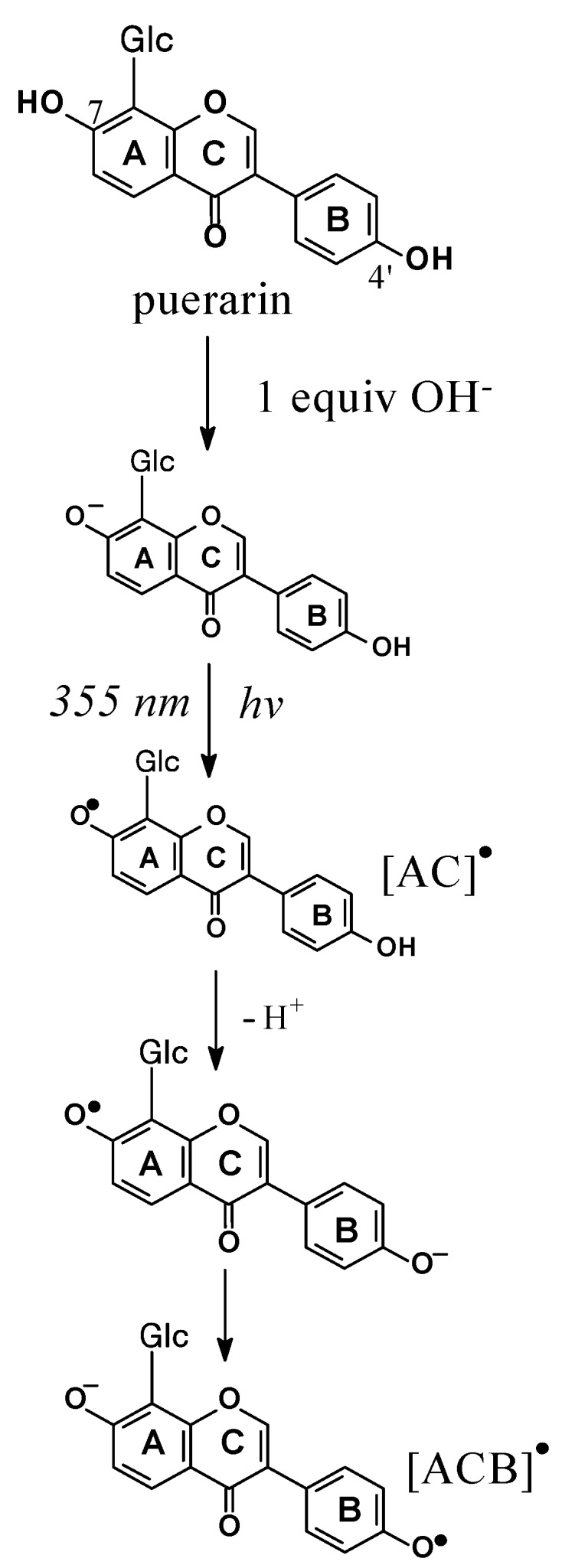
Mechanism of radical generation and rearrangement via photoionization of puerarin monoanion. The initially formed radical is a stronger acid than the ground state anion [26].

Direct photoexcitation of puerarin, a C-glycoside of the isoflavonoid daidzein (Scheme 1), in alkaline solution has been used to photooxidize puerarin and to yield the corresponding radicals [26]. The observed puerarin radicals such as the AC and the ACB radicals shown in Scheme 4 formed from puerarin anion were found to be insensitive to the presence of oxygen. Transformation of different puerarin radicals in the isoflavonone backbone occurred fast and was found independent of the concentration of parent molecules, and accordingly proposed to result from intramolecular electron transfer. Such remarkable intramolecular electron transfer of isoflavonones may explain the higher capacity in inhibition of lipid peroxidation for some isoflavonones compared to flavonones and even compared to quercetin, known as the most potent antioxidant in the flavonoid family in liposome and low-density lipoproteins [27]. For gallocatechins and catechins, which are constituents of green tea, as well as for rutin, inefficient coupling between radicals of the flavonoids A-ring and the unpaired electron formed from scavenging reactive radicals are in agreement with this suggestion [23,28].

*Structure-reactivity relationships*. Kinetic studies of reactions of flavonoids with active free radicals (N_3_^•^, ^•^OH, O_2_^•−^, *t*-BuO^•^, ArO^• ^and LOO^•^) in homogeneous solution using pulse radiolysis and laser flash photolysis combined with theoretical calculations have related the number and position of hydroxyl groups and the extension of conjugation to the efficiency of flavonoids as antioxidants [22,23,24,29].

Three structural requirements seems important: (i) the *ortho*-dihydroxy (catechol) structure in the B-ring, increasing the stability of oxidized flavonoid radicals through H-bonding or electron-delocalization; (ii) the 2,3-double bond, in conjugation with the 4-oxo function, enhancing electron-transfer and radical scavenging through electron-delocalization; (iii) the presence of both 3- and 5-OH groups, enabling the formation of stable quinonic structures upon flavonoid oxidation. A typical flavonoid which meets the above three criteria is quercetin, showing the highest antioxidant capacity. Aside from these structural requirements, the number and position of hydroxyl substituents on the flavonoid molecule, the presence of glycosides, and the overall degree of conjugation are important in determining their activities.

Recent measurements on the scavenging kinetics of the 2,2′-azino-bis(3-ethylbenzothiazoline-6-sulfonic) acid radical cation (ABTS^•+^) for the isoflavonones puerarin and daidzein by using stopped-flow spectroscopy comparing the 7- and 4′- phenol propyl derivatives indicate that the radical scavenging potential of the 4′-hydroxyl is twice as efficient as that of the 7-hydroxyl, and that the difference increases upon deprotonation [30,31]. In addition, despite the subtle structural differences, the isoflavonone genistein was also found twice as efficient in ABTS^•+^ scavenging and more efficient in inhibiting lipid peroxidation than the isomeric flavonone apigenin [32]. Based on quantum mechanical calculations, a low dipole moment and a large deviation from the A-to-B dihedral angle seem important for a high antioxidant efficiency for (iso)flavonoids.

### 2.2. Carotenoids

Epidemiological studies indicate that a high intake of carotenoids is beneficial to human health, an effect often assigned to the antioxidant activities of carotenoids [33,34,35], however, the molecular mechanism remains uncertain. To gain an insight at the molecular level, the kinetic aspects of the reaction of carotenoids with oxidizing free radicals seem essential especially for explaining the anti- or pro-oxidative effects of carotenoids. 

*Radical scavenging.* The reaction of carotenoids as scavengers of both short-lived and long-lived oxidizing radicals has been investigated. In homogeneous solutions reaction pathways have been found to depend on both the nature of the reacting free radical and the structure of carotenoid [18,36,37,38,39,40]. Three possible mechanism, shown as the reactions of Equations (6), (7) and (8), may be involved:


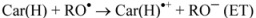
(6)


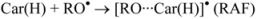
(7)


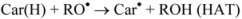
(8)

**Figure 1:**
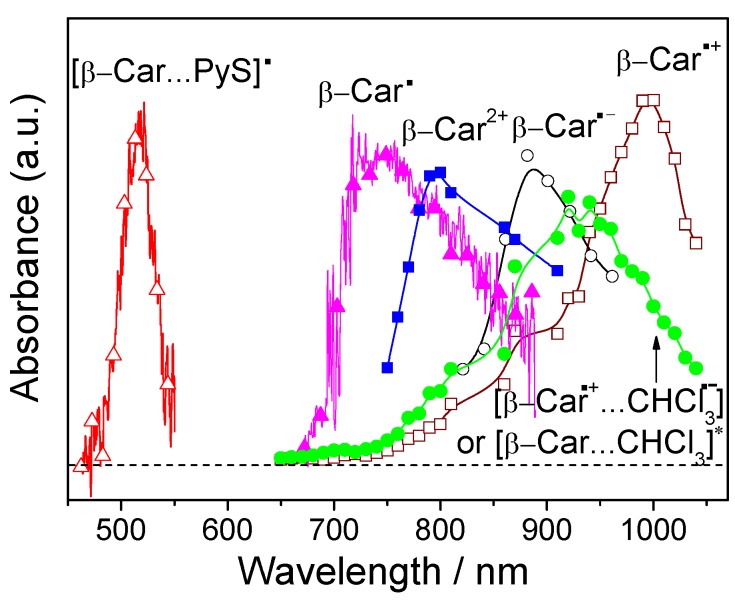
Absorption spectra of β-carotene (β-Car) oxidized or reduced radicals (*from left to right*) including adduct radical ([β-Car···PyS]^•^, ∆) [37], neutral radical (β-Car^•^, ▲) [37], dication (β-Car^2+^, ■)[43], radical anion (β-Car^•–^, ○) [42], ion-pair or exciplex ([β-Car^•+^···CHCl_3_^•^^−^]or [Car···CHCl_3_]*, ●) [43], and radical cation (β-Car^•+^, □) [43].

Many well-documented examples of the reaction mechanisms corresponding to the reactions of Equations (6) and (7) are known. Reaction with NO_2_^•^^+^ involves electron transfer (ET) to generate the β-carotene radical cation, while RS^•^ reacts by radical adduct formation (RAF). RSO_2_^•^ radicals undergo both ET and RAF in an approximate 3:1 ratio [36]. Hydrogen atom transfer (HAT) to yield the carotenoid neutral radical Car^•^ has been shown for reaction of β-carotene with ^•^OH as generated from the “photo-Fenton” reagent N-HPT (N-hydroxypyridine-2(1H)-thione) by using laser flash photolysis [37]. Absorption spectra of the three types of oxidized β-carotene radicals are shown in Figure 1.

The electron uptake for carotenoids scavenging superoxide radical anion O_2_^•−^ has firstly been confirmed under experimentally non-radiative conditions [38]. The detailed reaction of carotenoids with O_2_^•−^, further confirmed by theoretical calculations and experimental observations, seems different as two possible pathways [39,40,41]:


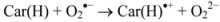
(9)


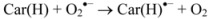
(10)

The first reaction is an electron transfer from the carotenoid like in the reaction of Equation (6). The carotenoid is oxidized by O_2_^•−^ to form the corresponding radical cation. The second reaction defines a novel antiradical activity of carotenoids taking up electrons, as the carotenoid is being reduced to a radical anion by O_2_^•−^. The absorption spectra of the radical anion Car^•−^ has been characterized using pulse radiolysis detection as shown in Figure 1 [42]. Astaxanthin seems to be a better O_2_^•−^ quencher than other carotenoids due to oxidation rather than reduction of O_2_^•−^.

*Stability of carotenoid radicals.* β-Carotene acts as a pro-oxidant at high oxygen pressures and high carotenoid concentrations, but as an antioxidant at low oxygen pressure. Such different behaviours of β-carotene and other carotenoids may rely on the stabilities of the carotenoid radicals formed from scavenging other radicals. Carbon-centred radicals are known to react readily with oxygen, giving peroxyl radicals, ROO^•^, which are generally more oxidizing than R^•^. The reactivity of the carotenoid radicals in secondary reactions is accordingly important in order to understand the efficiency of specific carotenoids as antioxidants.

The carotenoid radicals produced by radical scavenging react differently with oxygen depending on the nature of the radical scavenged. Carotenoid radical cations (Car^•+^) as produced in the reaction of Equation (6) through electron transfer are in general found unreactive under biologically relevant conditions as it normally does not react with molecular oxygen, but decays through dismutation to generate the dication Car^2+^ shown in Equation (11) and Figure 1 [43]: 


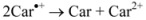
(11)

**Scheme 5:**
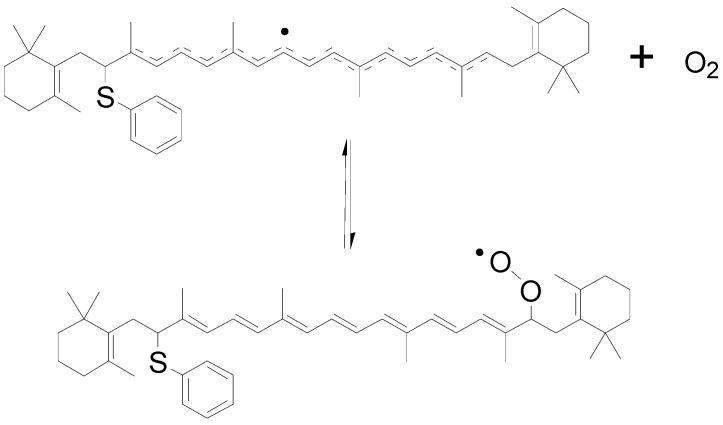
Proposed reversible addition of oxygen to carotenoid-derived carbon-centered neutral radical [44].

Another kind of carotenoid radicals, formed from direct free radical addition to the polyenic chain as shown in the reaction of Equation (7), is resonance stabilized and lack reactivity towards oxygen. 7,7′-Dihydro-β-carotene (77DH) or β-carotene adducted with the phenylthiyl radical (PhS^•^) is favoured by the extensive conjugation and the resultant resonance-stabilized radical shown in Scheme 5 [44]. The addition of oxygen to this carotenoid-derived carbon-centred neutral radical, apart from the substitution of phenylthiyl group, is structurally similar to the carotenoid neutral radical Car^•^ formed in the reaction of Equation (8), and the reaction is reversible. Kinetic data also indicate that the neutral radical Car^•^ is quenched by oxygen, forming a peroxyl carbon-centred radical.

*Photo-induced radical formation*. Carotenoids are good radical quenchers in their ground states, depending on their structures. Notably, the excited states are far better electron donors and may play important roles in radical reactions in the presence of light. The reactivities of carotenoid excited states as electron donors have accordingly been studied extensively.

Due to the C_2h_ symmetry of carotenoids, the optical transition from the ground state S_0_(1A_g_^−^) to the lowest singlet excited state S_1_(2A_g_^−^) is forbidden. The optially-allowed excitation to the S_2_(1B_u_^+^) excited state or other higher lying singlet excited states relax quickly (<200 fs) to the S_1 _state living in a few to a few tens of picoseconds. Despite the short lifetimes of carotenoid excited states including the S_2_(1B_u_^+^) and the S_1_(2A_g_^−^) states, as well as the lowest triplet excited state T_1_ typically living in microseconds, the electron-withdrawing chlorinated solvents allow the study of the electron donating capacity of carotenoids in these excited states.

The electron transfer from carotenoid singlet excited states to solvent yielding Car^•+^ occurs in the femtosecond time domain [45]. The lowest triplet excited state of β-carotene and lycopene has been found to be a precursor of the radical cation in chloroform, and the reactivities of four carotenoids in Scheme 2 were studied systematically [46,47,48]. For the singlet excited states investigated by subpicosecond time-resolved absorption spectroscopy in combination with spectroelectrochemical determination of the NIR absorption of Car^•+^, the S_2_(1B_u_^+^) state was concluded to eject electrons directly into chloroform leading to the rapid formation of Car^•+^ in a time scale of ~100 fs. However, the S_1_(2A_g_^−^) state was found inactive. On the other hand, Car^•+^ was also produced from the T_1_ state populated via anthracene sensitization. Because this state cannot directly transfer electrons to chloroform for energetics reasons, a solute–solvent complex absorbing around 900 nm was suggested to mediate the production of Car^•+^. An intermediate with similar absorption in the near infrared spectral region was proposed to be an ion-pair [Car^•+^···CHCl_3_^•^^−^] or an exciplex [Car···CHCl_3_]* as seen in Figure 1. Otherwise, the ground state of carotenoids was also found to react with a secondary solvent radical to yield Car^•+^. Among all carotenoids studied having 11 conjugated double bonds, zeaxanthin ranks with the highest reactivity in both forming Car^•+^ from the S_2_(1B_u_^+^) and the T_1_ states. However, fucoxanthin, a major marine carotenoid found in edible seaweeds, structurally including an allenic bond and a 5,6-monoepoxide, differs from the common carotenoids such as β-carotene and lycopene. In chloroform, fucoxanthin radical cation was mainly formed from the S_2_(1B_u_^+^) state, whereas in methanol it was mainly formed from the T_1_ state [49].

**Scheme 6:**
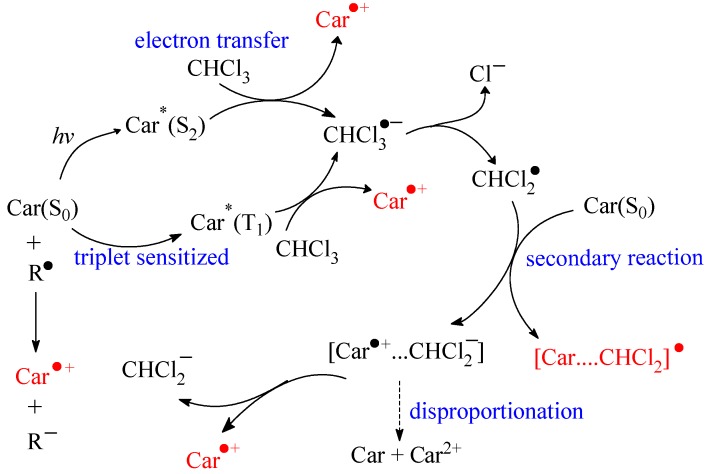
Proposed reaction mechanism of carotenoid radical formation following direct photo-excitation or triplet sensitization of carotenoids (Car) in chlorinated solvent (chloroform). A solvent radical can initiate further carotenoid radical cation formation [43].

A precursor absorbing at 720 nm for the β-carotene radical cation was suggested to be an ion pair from which the β-carotene radical cation is formed in neat chloroform, while in more polar solvents it reacts at least partly through disproportionation in a bimolecular reaction promoted by the presence of ions [43]. The precursor for β-carotene radical cation decays in a second-order reaction in the mixed solvents, with a rate decreasing for increasing dielectric constant of cosolvents. The detailed mechanistic description on Car^•+^ production from carotenoid singlet and triplet excited states are summarized in Scheme 6.

*Structure-reactivity relationship*. Structure-reactivity relationship of carotenoids scavenging free radicals *in vitro* is well established, using nanosecond laser flash photolysis measuring the relative rates of carotenes’ and xanthophylls’ reaction with *tert*-butoxyl radicals [50]. Canthaxanthin and β-apo-8'-carotenal hardly reacted, and astaxanthin did not react at all. The carotenoid radicals formed are rather long-lived (second-order decay on a millisecond time scale). Lycopene, with the characteristic lower energy of the near-infrared transition for its radical cation, has been concluded to be a more efficient antioxidant.

## 3. Flavonoid Interactions with Carotenoids in Heterogeneous Systems

In heterogeneous systems such as liposomes or more complicated real food systems, different antioxidants will be distributed differently owing to their different lipophilicities. The radical scavenging capabilities of both flavonoids and carotenoids will accordingly be modulated depending on the microenvironment, and the results obtained for such heterogeneous and more realistic systems may be difficult to be understood merely based on the results in homogeneous solutions.

The interaction of antioxidants with lipid membranes is of special interest as the distribution and orientation of antioxidants in the heterogeneous system not only affect the antioxidant itself, but also modify the structure and the property of the membrane. Such effects may even be more pronounced for mixture of different types of antioxidants. The effectiveness of carotenoid as antioxidant in protecting the membrane against ROS damage thus depends upon their interaction with vitamins E and C as well as with flavonoids and even with other carotenoids. 

### 3.1. Heterogeneous Systems with Flavonoids and Carotenoids

**Scheme 7:**
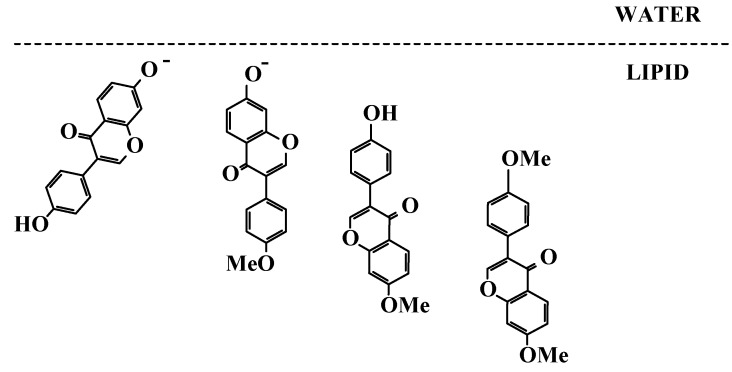
Proposed deposition and orientation of daidzein and different anisol daidzein derivatives in the phosphatidyl cholin bilayer of liposomes.

*Flavonoids*. The membrane stiffness may be probed by fluorescence spectroscopy as shown for liposomes by the use of the fluorescent probes 8-anilino-1-naphthalenesulfonic acid (ANS) and 16-(9-anthroyloxy) palmitic acid (16-AP), which distribute preferentially at the surface and in the interior of the bilayer, respectively [31]. Daidzein and anisole derivatives of daidzein significantly lowered membrane fluidity, in effect hampering the radical mobility and decreasing the rate of oxidative damage. Structural comparison further indicated that deprotonation of 7-OH in A-ring enables daidzein to interact with the polar head of phosphatidyl cholin and, therefore, to approach the membrane surface as illustrated in Scheme 7. The isoflavone B-ring locating in the hydrophobic domain of the bilayer restricts the diffusion of free radicals and scavenges lipophilic radicals. Clearly this is different from homogeneous solution where the 4′-OH in the B-ring is the key functional group for radical scavenging. In liposome both the modification of membrane fluidity upon flavonoid deposition and the radical scavenging capacity of flavonoid alleviate the oxidative damage, whereas in homogeneous solution only direct radical scavenging is of importance.

*Carotenoids*. The effect of carotenoids on structural and dynamic properties of biological membranes and model lipid membranes has been extensively studied using several experimental techniques: electron paramagnetic resonance (EPR), light scatterings in liposome suspensions, differential scanning calorimetry, X-ray diffractometry and nuclear magnetic resonance of phospholipid molecules [51,52,53,54].

**Scheme 8:**
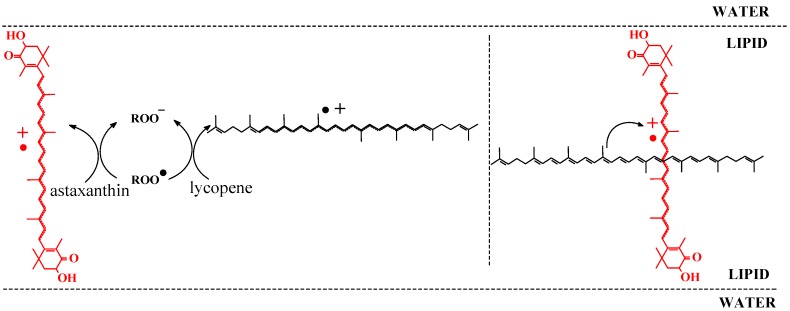
Respective radical scavenging mechanism and cooperation of astaxanthin and lycopene as antioxidants in liposomal membrane.

The ability of dietary carotenoids acting as antioxidants in such membrane systems is dependent on a number of factors, especially on the length of the conjugated double bond system and the presence of keto and hydroxyl groups affecting how these molecules modify the membrane fluidity. Carotenoid hydrocarbons tend to locate parallel with the membrane surface within hydrophobic membranes, whereas xanthophylls having polar end groups tend to adopt a rigid, membrane-spanning orientation as shown in Scheme 8 [31,55,56]. Carotenoids accordingly stabilize the membrane depending on their structures.

### 3.2. Carotenoid and Vitamin Antioxidants

The interaction between carotenoids and vitamins C and E is important for antioxidant synergism [19,20,57,58], since^.^carotenoids may be regenerated from their radical cations as in the reaction of Equation (12), where a tocopherol (TOH) homologue regenerates a carotenoid: 


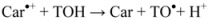
(12)

Real time spectroscopic detection in DPPC (dipalmitoyl phosphatidyl choline) liposome indicates that while the parent hydrocarbon carotenoid almost completely resides in the non-polar region of the liposome, the carotenoid radical cation can be repaired by a water-soluble ascorbate (AscH^−^) at the water/lipid interface:


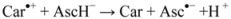
(13)

Regeneration of α-tocopherol from the one-electron oxidized form by the more reducing ascorbate is well-established at water/lipid interfaces. β-Carotene is now suggested to be the primary antioxidant at least under some conditions. α-Tocopherol may then reduce the β-carotene radical cation according to the reaction of Equation (12). 

**Scheme 9:**
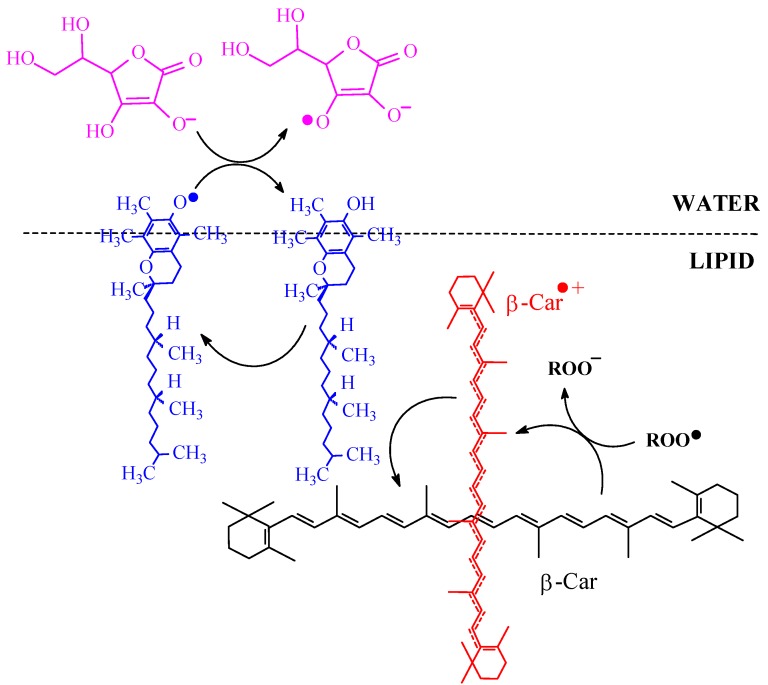
Proposed mechanism of synergistic antioxidation interactions between β-carotene (β-Car), vitamins C and E.

The β-carotene radical cation with a positive charge and higher polarity than the lipophilic parent molecule tends to rotate and move towards the more polar interface after scavenging lipid peroxide radicals, which facilitate the regeneration of β-carotene radical cation by α-tocopherol. α-Tocopherol in turn can be regenerated by ascorbate in the water/lipid interface, as depicted in Scheme 9.

### 3.3. Interactions among Carotenoids

The orders of ease of electron transfer among carotenoids in homogeneous solution have been well established using pulse radiolysis [19]. Lycopene is the strongest reducing agent and accordingly the most easily oxidized, whereas astaxanthin is the weakest reducing one. The more oxidizing radical cations of astaxanthin or canthaxanthin may be reduced by lycopene, by β-carotene or by zeaxanthin.

Different carotenoids show synergism as antioxidants in heterogeneous systems due to their different lipophilicity. Such antioxidation synergism was recently verified for astaxanthin combined with β-carotene or with lycopene for the inhibition of oxidation initiated by 2,2’-azobis(2,4-dimethylvaleronitrile) (AMVN) in liposomes. The mechanism behind seems to depend on the spatial distribution of carotenoids as illustrated in Scheme 8 for the combination of astaxanthin and lycopene. Astaxanthin, anchored in the lipid/water interface, is oxidized by the initiating AMVN radicals to yield the radical cation in competition with oxidation of PC. The unpaired electron in the radical cation of astaxanthin will relocate into the centre of the polyene chain of carotenoid due to resonance stabilization [55,59]. The polyene chain in the lipophilic centre of the membrane may abstract an electron from the more reducing carotenoid lycopene or β-carotene in effect regenerating astaxanthin at the expenses of lycopene or β-carotene as demonstrated directly by electrospectroscopy. Astaxanthin accordingly acts as a wire to facilitate the flow of electron from the more reducing carotenoid in the inner of the membrane to the interface where oxidation is initiated. Zeaxanthin and astaxanthin do not act synergistically as antioxidants under the same conditions, as they have a similar spatial distribution in the membrane.

### 3.4. Flavonoid Interaction with Carotenoids

**Scheme 10:**
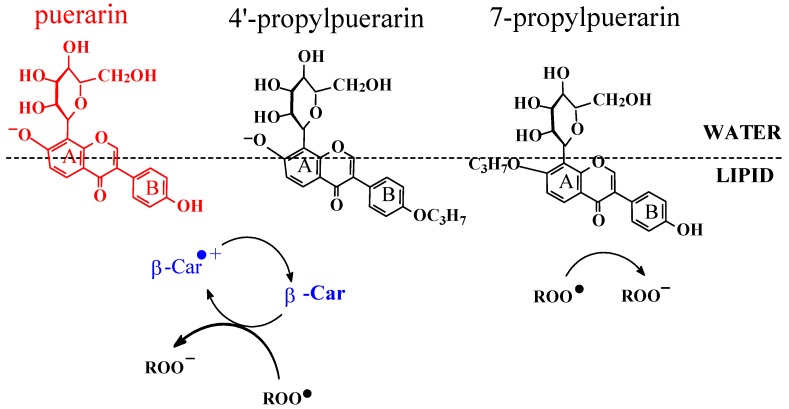
Proposed mechanism of synergistic antioxidant interaction between β-carotene and puerarin in liposome. Synergism is seen for 4′-propylpuerarin, where β-carotene (β-Car) is regenerated at the interface by 7-phenolate group. 7-Propylpuerarin is more lipophilic and less acidic and becomes an antioxidant in the lipid phase [60].

The interactions between β-carotene and a series of flavonoids in liposomes have now been investigated systematically. β-Carotene and the isoflavonone puerarin were found to show a clear synergism in phosphatidyl choline liposomes measured as prolongation of the lag phase for the formation of conjugate dienes as primary lipid oxidation products [25]. The interaction of β-carotene with puerarin derivatives with one of the two phenolic groups blocked demonstrated the synergism for β-carotene and the A-ring phenolate (Scheme 10), but not for the B-ring phenolate [60]. Fast regeneration of β-carotene from the radical cation Car^•+^ by the 7-phenolate of puerarin (Pue) explains the antioxidant synergism:


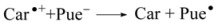
(14)

The 4΄-phenolate is less acidic, and it penetrates into the lipid phase and shows no antioxidation synergism with β-carotene. The rate constants for such regeneration reactions between carotenoid radical cations and isoflavonones have now been determined for β-carotene, zeaxanthin, astaxanthin and canthaxanthin in combinations with flavonoid molecules, and been found to depend on carotenoid structures as shown for dianions of daidzein and puerarin [21]. The presence of a keto group as in astaxanthin or canthaxanthin was concluded to facilitate the electron transfer making the regeneration more efficient.

Interaction between β-carotene and a series of (iso)flavonoids (glycosides) as seen in Table 1 have been shown to lead to antioxidant synergism, and three factors responsible for a higher antioxidant synergism have been identified: (i) appropriate distribution of (iso)flavonoids at the lipid/water interface, (ii) (iso)flavonoids or anionic form being more reducing than the carotenoids, (iii) fast electron transfer reaction from (iso)flavonoid or anion to carotenoid radical cation [61,62]. The water/lipid partition of the (iso)flavonoid is of particular importance for the contact to the carotenoid radical cation. 

**Scheme 11:**
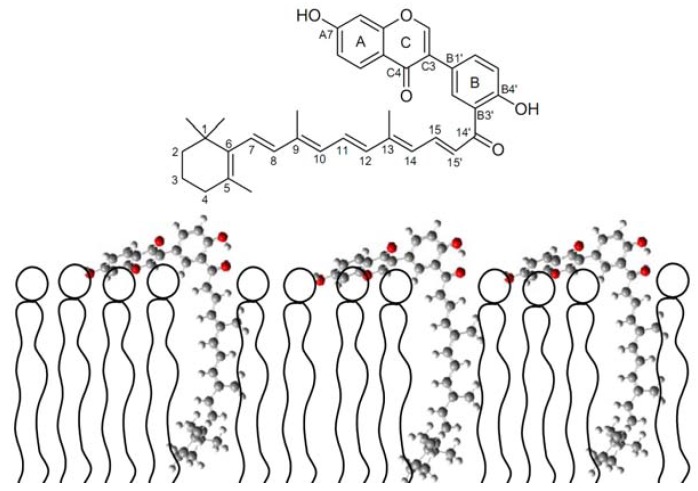
Schematic model of retinylisoflavonoid deposition in liposome [64].

**Table 1:** Efficiency of antioxidant synergism (SE, positive) or antagonism (AE, negative) in percentage between β-carotene and a number of (iso)flavonoids and their glycosides [61,62,65].

sample	daidzein	puerarin	baicalein	baicalin	quercetin	rutin	EC	EGC	ECG	EGCG
**SE** **or AE (%)**	−29	47	−20	0	52	32	−62	−30	−52	−48

However, the dipole moment of the (iso)flavonoid as a microscopic property available from quantum mechanical calculations, rather than the partition coefficient as a macroscopic property, seems important for control of the electron transfer from the (iso)flavonoid in water/lipid interface [32,63]. This antioxidant synergism is based on a bimolecular reaction. Linking the polyene of the carotenoid directly with the (iso)flavonoid as in the synthetic antioxidant molecule retinylisoflavonoid shown in Scheme 11, resulted in a higher antilipooxidation activity in protecting membrane lipids than the parent compounds alone or in combination most likely due to a more rapid intramolecular electron transfer [58,64].

Antioxidant synergism between carotenoids and flavonoids depends, however, strongly on the structure of the flavonoids. β-Carotene even showed antioxidant antagonism with the very reducing green tea polyphenols (−)-epicatechin (EC), (−)-epigallocatechin (EGC), (−)-epicatechin gallate (ECG) and (−)-epigallocatechin gallate (EGCG) under conditions where both β-carotene or the tea polyphenols alone were active as antioxidants [66]. This antagonism, clearly not predicted by the linear free energy relationship establish for regeneration reactions of carotenoids by flavonoids is unclear yet, but may indicate the existence of an “inverted region” for electron transfer.

## 4. Future Trends

Real-time radical kinetics, providing understanding of reaction mechanisms behind synergistic and antagonistic effects of combinations of natural antioxidants, should provide for a more rational design of protective systems for oxidation-sensitive biological structures including both lipids and proteins. Such time-resolved studies of bimolecular radical scavenging reactions in the micro- and nanosecond time domains, and of unimolecular radical rearrangements with subpicosecond time resolution supported by quantum mechanical calculations, may gradually replace the determination of “antioxidant capacity” in traditional antioxidant assays, and replace more macroscopic description of “antioxidant activity” as a measure of rate of antioxidative action.
